# Relation between two evolutionary clocks reveal new insights in bacterial evolution

**DOI:** 10.1099/acmi.0.000265

**Published:** 2022-02-16

**Authors:** Gur Sevillya

**Affiliations:** ^1^​ Faculty of Biology, Technion - Israel Institute of Technology, Haifa, Israel

**Keywords:** bacterial taxonomy, bacterial evolution, genome rearrangement, gene order, gene distance, HGT, synteny

## Abstract

New insights in evolution are available thanks to next-generation sequencing technologies in recent years. However, due to the network of complex relations between species, caused by the intensive horizontal gene transfer (HGT) between different bacterial species, it is difficult to discover bacterial evolution. This difficulty leads to new research in the field of phylogeny, including the gene-based phylogeny, in contrast to sequence-based phylogeny. In previous articles, we presented evolutionary insights of Synteny Index (SI) study on a large biological dataset. We showed that the SI approach naturally clusters 1133 species into 39 cliques of closely related species. In addition, we presented a model that enables calculation of the number of translocation events between genomes based on their SI distance. Here, these two studies are combined together and lead to new insights. A principal result is the relation between two evolutionary clocks: the well-known sequence-based clock influenced by point mutations, and SI distance clock influenced by translocation events. A surprising linear relation between these two evolutionary clocks rising for closely related species across all genus. In other words, these two different clocks are ticking at the same rate inside the genus level. Conversely, a phase-transition manner discovered between these two clocks across non-closely related species. This may suggest a new genus definition based on an analytic approach, since the phase-transition occurs where each gene, on average, undergoes one translocation event. In addition, rare cases of HGT among highly conserved genes can be detected as outliers from the phase-transition pattern.

## Introduction

Two main processes influence the bacterial genome – point mutation of nucleotides and recombination of large pieces of DNA (here we use the term recombination as the general case of translocation or genome rearrangement, or any other change in gene order) [1], and the relation between these two processes is the target for experimental and theoretical investigation. Point mutation is the process where a single nucleotide base is changed, inserted, or deleted from a DNA sequence, and it usually occurs during DNA replication. The term ‘mutation rate’ refers to the frequency of new point mutations in a single gene or organism over time [2]. The evolutionary theory of mutation rates, identifies three principal forces involved: the deleterious mutations with higher mutation, the advantageous mutations with higher mutation, and the metabolic costs and reduced replication rates that are required to prevent mutations. According to this, although higher mutation rates enable a better adaptation, an excessive mutation rate might lead to an ‘error catastrophe’, the extinction of an organism (often in the context of micro-organisms) [3]. Horizontal gene transfer (HGT) is one of the major recombination processes, and it is the movement of genetic material between organisms rather than by the vertical transmission of DNA from parent to offspring. In general, recombination, or more specifically HGT, is the mechanism of changing the genetic material, in a large scale of the genes. There are three main processes involved in HGT of bacteria genome: (1) conjugation, which takes place through a tube between the two cells of bacteria; (2) transformation, which is a kind of genetic recombination where only the carrier of genes, i.e. the DNA molecules of donor cell, pass into the recipient cell through the liquid medium; and (3) transduction, which is a special method of genetic recombination where genetic material is transferred from the donor to the recipient cell through a non-replicating bacteriophage. HGT is an important factor in the evolution of micro-organisms and has a great influence on the phylogenetic tree, as it turns it to be a network of transmitted genes across species [4]. Gene order is the permutation of genome arrangement, and this measure is influenced by the HGT process [5]. To simplify matters, we will regard the HGT as the main cause of gene order, although there are other factors that influence gene order, which are equivalent in the computational point of view.

The term ‘molecular clock’ is used to describe the mutation rate of biomolecules and deduce the time since divergence. This technique is an important tool in molecular systematics. The biomolecular data used for such calculations is usually DNA or protein sequence, or gene distance, as it is often called. This relies on the genetic equidistance phenomenon, the concept that sister species are approximately equidistant to a simpler outgroup as measured by DNA or protein dissimilarity, even if the mutation rate is not constant [6]. Nevertheless, it is accepted that five factors combine to limit the application of molecular clock models: changes in generation times, population size, species-specific differences, studied protein function and the intensity of natural selection [7]. Therefore, the ‘relaxed molecular clock’ model was established to improve clock accuracy [8]. However, at very short time scales, many differences between samples do not represent fixation of different sequences in the different populations. This leads to a potentially dramatic inflation of the apparent rate of the molecular clock at very short time scales [9]. In this work, we will try to harness gene order as an evolutionary signal to create a more accurate clock for closely related species.

In addition to the theoretical aspects of evolution in light of gene order and gene distance, in this paper we will discuss two main practical problems: detection of HGT among the 16S gene and the species problem.

As stated above, HGT is an important phenomenon responsible for genome dynamics in bacteria and plays an important role in adaptation and selection [10]. According to Woese’s ‘complexity hypothesis’, the 16S gene tends not to undergo HGT, and as a result, this gene has been selected as a gold standard marker gene for prokaryotic classification [11]. Recent studies show that this gene, and other housekeeping genes, might have undergone HGT events in some cases [12–15]. Such cases of variations in 16S gene distance among closely related species is known, for example among the *Chlamydia* and *Thermoanaerobacter* genus [15]. This phenomenon has an ecological, evolutionary and taxonomic importance, and hence the importance of detection of such cases of HGT of the 16S gene.

Currently, there are two prevailing approaches for detecting HGT, the phylogeny-based and the composition-based approach (also called ‘parametric approach’). The phylogeny-based approach takes a relatively large set of homologous coding sequences (originating from a common ancestor), constructs their corresponding phylogeny, and contrasts it with the phylogeny of the original species [16]. The composition-based approach contrasts genomic sequences of different compositional structure such as G+C content, dinucleotide frequencies, or codon usage biases, striving to infer different origins [17]. Another approach offered by our group, is based on synteny index, a gene order heuristic, and is recommended especially for closely related species [18]. All these methods lack the ability to detect precisely HGT events among housekeeping genes such as the 16S gene, due to lack of signal. Here we offer a new approach for detection of HGT of the 16S gene, based on the relation between the gene distance-based clock and the gene order-based clock.

According to ‘The species problem’, (or ‘The grouping problem’, as it is sometimes referred to in the literature), species within a genus are supposed to be somehow similar, but there are no objective criteria for grouping species into genera [19], since genus definition is not based on an analytical measurement and it is subject to bias according to the researchers’ background. Although 16S-based phylogeny is arguably excellent for classification of Bacteria and Archaea from the domain level down to the family or genus, it lacks resolution below that level [20]. An operational taxonomic unit (OTU) is an operational definition used to classify groups of closely related individuals, and it refers to clusters of organisms, grouped by DNA sequence similarity of a specific taxonomic marker gene [21]. In other words, OTUs are pragmatic proxies for microbial ‘species’ at different taxonomic levels, in the absence of traditional systems of biological classification as are available for macroscopic organisms. Still, this approach is lacking theoretical basis since there is none used to determine the threshold value (which is set, arbitrarily, at 98.7 % for species, 95 % for genus [22]). Another recommendation to delineate species is using a 70 % DNA–DNA binding criterion [23], but this approach also does not correspond to a theory-based concept of what properties a species should have, and it is calibrated empirically to yield many of the phenotype-based species already recognized at the time of its inception [24]. Here, based on the relation between the two clocks, we offer an innovative solution for the grouping problem.

In this work, we use of the term ‘Synteny Index’ (SI) first defined in our pilot work [25] to measure the evolutionary divergence between organisms based on gene order. Here, we harness the SI approach to solve the four problems mentioned above. First, we present a relationship between recombination (i.e. gene order) and mutation (i.e. gene distance). We separate this into two parts: one among closely related species, where we found a constant, approximately linear, relation. This local relation we called point mutation to HGT ratio (‘PMTH ratio’). The other expresses the global ratio (i.e. not only between closely related species) between recombination and point mutation, which is considered a phase transition shape, such that for closely related species there is very low point mutation evolutionary signal, and it turns over sharply at a specific point. This finding of the phase transition pattern leads to the ‘evolutionary scale concept’, in which the evolutionary time since speciation can be presented in a linear format, separated into two sections – the closely related species section, characterized by strong gene order signal, and the non-related species section characterized by strong point mutation signal. These theoretical findings (PMTH, phase transition, the evolutionary scale) lead us to practical solutions for two important problems within microbiology, and these are the aims of this work: the first one is suggestion of a new definition of the genus concept, and we found that the phase transition point may serve as a border line between genera. The second is the question of HGT events of the 16S gene. We found outliers above the phase transition trend line, which count for about 1 % of the data, analysed these outliers and we suggest at least part of these outliers represent rare events of HGT of the 16S gene.

## Methods

### SI definition

The Jaccard index is a common statistic used to compare between two sets, defined as the size of the intersection of the sets divided by their union, $J\left(A,B\right)=\left|\frac{A\cap B}{A\cup B}\right|$ . Inspired by the Jaccard index, the Synteny Index (SI) measures the phylogenetic relationship between two genomes, based on their genes sequence as opposed to the DNA (or protein) sequence, as in traditional phylogenetics. The Jaccard index is used here among neighbourhoods of genes. Specifically, for a gene $g_{0}$ residing in a genome A , we denote $N_{k}\left(g_{0},A\right)$ a 2k -neighbourhood of $g_{0}$ in A , i.e. the k genes upstream and k genes downstream to $g_{0}$ in the genome. Assume $g_{0}$ resides also in genome B , then the syntenyindex(SI) of $g_{0}$ with respect to genomes A and B , ${SI}_{k}(g_{0},A,B)$ or just ${SI}_{k}(g_{0})$ , is the relative (normalized) number of common genes in both neighbourhoods $N_{k}(A,g_{0})$ and $N_{k}(B,g_{0})$ , or formally $\frac{1}{2k}\left|N_{k}(A,g_{0})\cap N_{k}(B,g_{0})\right|$ . If $g_{0}$ does not belong to A or B we set ${SI}_{k}\left(g_{0}\right)=0$ . The average SI between A and B , denoted ${\overset{-}{SI}}_{k}(A,B)$ (or simply $\overset{-}{SI}$ when it is clear), is obtained by averaging ${SI}_{k}\left(g_{0},A,B\right)$ for all genes $g_{0}$ residing either A or B . $\overset{-}{SI}$ represents the evolutionary relation between the genomes as it indicates on the HT activity between them. By its definition $\overset{-}{SI}$ represents similarity. Hence, similarly to Jaccard distance, which measures dissimilarity between samples sets, defined $d_{J}\left(A,B\right)=1-J(A,B)$ , we also convert the $\overset{-}{SI}$ to a distance measure, by subtracting it from 1. In order to be consistent, along this paper we will refer to the distance index, not to the similarity index. The above regards pairwise genome distance. On the level of species set, the $\overset{-}{SI}$ -based phylogenetic approach receives as an input a set of genomes, each in a format of a gene-list, and returns a distance matrix as in output. Each entry in the matrix holds the pairwise $\overset{-}{SI}$ distance between the respective two genomes.

### EggNog database

We used the eggNOG (version 3) database [26] as source for the gene order of species. The eggNOG database contains 1133 species, most of them bacteria. The database provides the species proteins sequences in FASTA files format, and genes are clustered intoCluster of Orthologous Groups (COGs). Based on the proteins sequences and the COG system provided by eggNOG, we created for each species a COG-file, i.e. the file contains the COG names for each gene in the genome in the same order as it appears in the genome. These COG files are used as input files for the SI method.

### RDP database

In order to calculate gene distance between species, we used the 16S gene from The Ribosomal Database Project [27] (RDP) database. We calculated the intersection group between eggNOG species and RDP species, and extracted the relevant sequence of the 16S gene.

### Species clustering

In order to cluster all eggNOG species into some groups of closely related species, we executed the following approach, as described in [28]. We aimed to find cliques of related species based on the SI distance matrix, i.e. groups of species in which each organism relates to all the other clique members in less than some threshold τ . For that, we created a graph object where the species are the nodes ( V ) and the edges ( E ) connect between nodes so there is an edge between two nodes if their SI distance is below the threshold τ . By that, we got the graph $G_{\tau }(V,E)$ . A clique in this graph is a subset of nodes such that every two nodes in the subset are adjacent (connected). While finding all cliques in a graph is a computationally intractable task, there are good heuristics for it. Hence, we executed a clique finding heuristic algorithm (based on *networkX* module for Python [29]) for iteratively extracting the largest clique. We took into consideration only cliques containing more than five species. We used 0.95 as the threshold value τ , which provides a meaningful clustering as well as meaningful phylogenetic results. The neighbourhood size for all SI calculations in this work was k=10 .

### Statistics

Data analysis, calculations and statistics in this work done using excel and python (scipy [30], sklearn [31], NetworkX [29]).

## Results

### Part 1: the relation between two evolutionary signals- *P*_HGT_ and *P*_PM_, among closely related species:

In a previous paper [28], we defined PMTH , to measure the linear ratio between point mutation process and genome rearrangement. In this study we dive into the depths of this relation. At first, we investigate the relationship between these two measurements (gene order and gene distance) among closely related species. For each pair of species of closely related species (cliques, as declared in ‘Species clustering’ of Methods) we calculate both SI and 16S gene distance. For 16S gene distance we used the Jukes Cantor model [32] for distance between sequences. For gene order we used the model developed in [33], which declares the expected SI value for a proper number of recombination events:

(1) $$E\left(SI_{k}\right)=\text{(}1-e^{-\;\frac{3-\frac{5k}{n-1}}{n}p}\text{)}\;(1-\frac{2k}{n-1})$$

where k is neighbourhood size, *n* is genome size and p is number of recombination events. We note that ${E\left(SI\right)}^{\infty }=1-\frac{2k}{n-1}$ is the expected SI after infinite number of translocation events (see [33] for proof). This model enables the translation of the observed SI to the expected number of recombination events:

(2) $$E(p)=-\frac{n}{3-\frac{5k}{n-1}}\ln(1-\frac{{\overset{-}{SI}}_{k}}{\left(1-\frac{2k}{n-1}\right)})$$

**Fig. 1.**
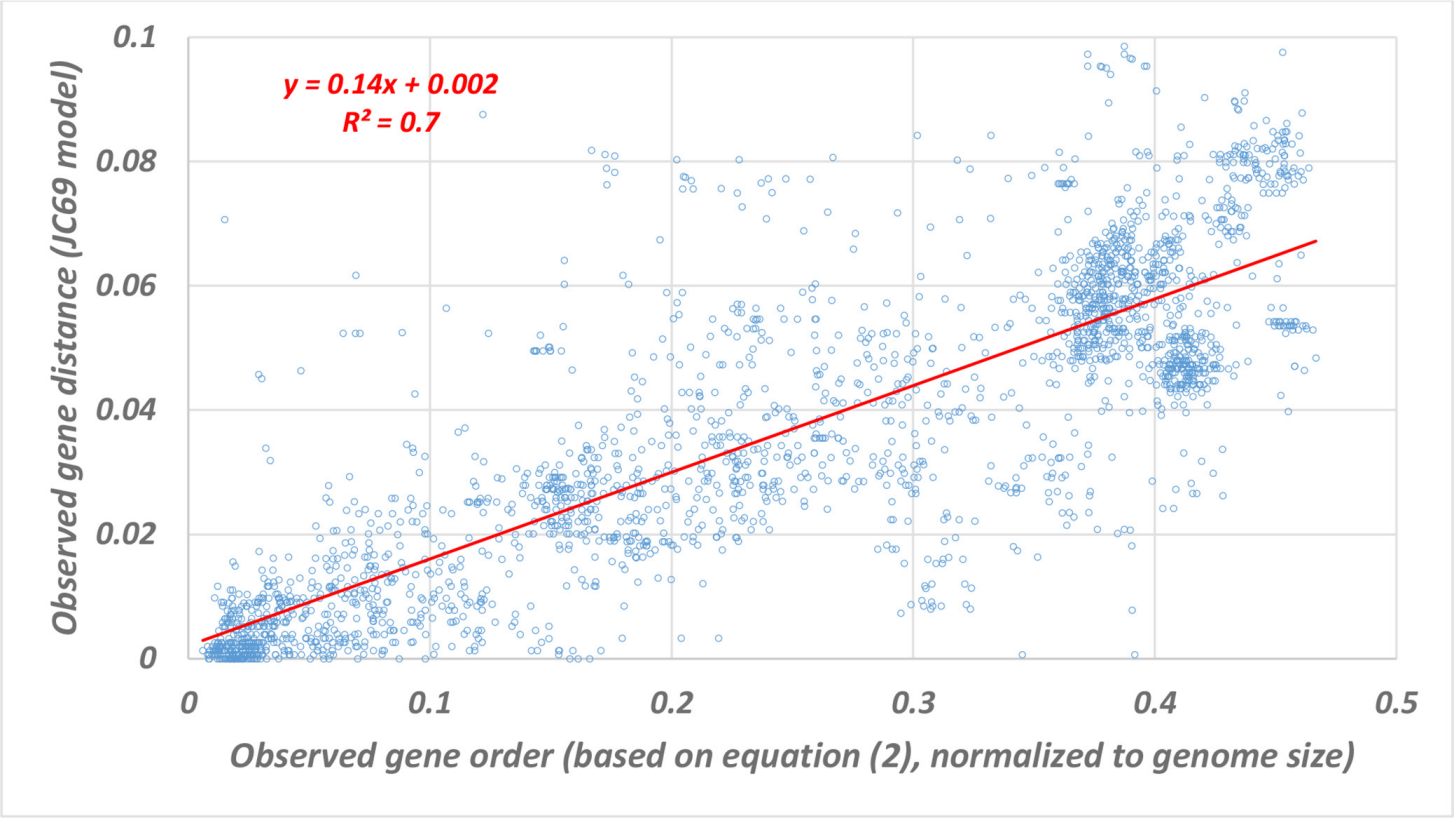
Linear relationship between gene order and gene distance for closely related species. Here we present the correlation between observed probability to point mutation (*P*_PM_, gene distance) to observed probability to gene rearrangement event (*P*_GR_), inside cliques. For each pair in each clique we calculate both *P*_PM_ (based on 16s gene distance from RDP repository) and *P*_GR_ and plotted these two measures for each pair in each clique. Outliers pairs with gene distance > 0.1 were eliminated (165 pairs, out of 2562 pairs). There is a significant linear correlation (red line) between these measures (*df*=2397, *R* ^2^=0.6995, *P*<0.0001, 95 % confidence interval for the coefficient 0.144-0.134).

Results are shown in Fig. 1. A nearly constant value of PMTH ratio is found among pairs of organisms of these closely related species, as reflected by the linear line in Fig. 1. Therefore, the gene order signal is about seven times ( $\frac{1}{0.14}≅7$ , where 0.14 is the coefficient factor of the regression) stronger than the point mutation signal, among closely related species ( $df=2397,\;R^{2}=0.7,\;p<0.0001$ , 95 % confidence interval 0.144–0.134). This finding is well consistent with the previous works mentioned above [34], but while these previous works focus on a small set of organisms, here we show this ratio is seeming to be uniform among wide variety of bacteria

### Part 2: The relation between two evolutionary signals- *P*_HGT_ and *P*_PM_, among non-closely related species

**Fig. 2.**
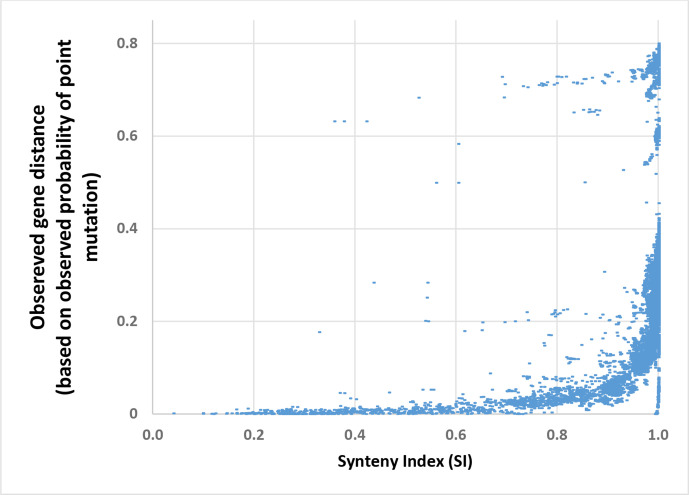
Point mutation proportions (*P*_PM_) in the 16s gene as function of SI signal. Each point represents pair of species appears both in eggNOG and RDP databases (49997 pairs in the graph). For each pair, we calculate both 16s and SI distances based on RDP and eggNOG databases, accordingly. A clear phase transition is evident in the relation between these two measures, so that *P*_PM_ increase marginally for low values of SI and this pattern changes sharply when SI≅0.9. We can say that for low SI values, the curve presents high level of elasticity in terms of $SI\;(E_{SI,P_{PM}}§gt;1)$ , and for high values of SI, the curve presents low level of elasticity in terms of SI $SI\;(E_{SI,P_{PM}}§lt;1)$ . We found that $E_{SI,P_{PM}}§gt;1$ when *SI*=0.925.

Next, we expanded our analysis to explore the relation between the point mutation process and the HGT process without the restriction of closely related species. We calculate the SI distance and the *$P_{PM}$* distance between each pair in intersection between the two databases (eggNOG and RDP) and plotted these two measures. In contrast to Fig. 1, here we didn’t use the estimation function from SI to probability of HGT because it is not reliable for such a wide range of SI’s values, especially where SI→1 . We present the results in Fig. 2. A phase transition pattern shows in the data, i.e. for low values of SI there is very slow increasing of $P_{PM}$ values, but where SI≅0.9 , the relations turn sharply. A similar pattern was published for a smaller dataset of archaeal genomes [35]. We note that the dataset contains ~50 000 points, but those data points do not distribute equally within the graph. SI values greater than 0.9 account for 96.6 % of the data and $P_{PM}$ values between 0.1 to 0.3 account for 87 % of the data (86.83 % data points fell into these two conditions of *SI* >0.9 and 0.1 <*P*_*PM*_ <0.3), because most of the pairs contain two species which are not closely related. Outliers are clearly can be seen in this graph and we will discuss this below. In order to analyse the phase transition phenomenon and detect the specific point of the phase transition, we use the elasticity term [36], $E_{SI,P_{PM}}$ , which is defined as

(3) $$E_{SI,P_{PM}}=\frac{dSI/SI}{dP_{PM}/P_{PM}}$$

This term gives the percentage change in SI quantity in response to a one percent change in $P_{PM}$ . Note that for $E_{SI,P_{PM}}=1$ , each change in SI case equal change in *P*_PM_. For $E_{SI,P_{PM}}§amp;lt;1$ , each change in SI case larger change in *P*_PM_, and when $E_{SI,P_{PM}}>1$ , each change in SI case lower change in *P*_PM_. According to this, we can separate the data into two sections. The first section, for $E_{SI,P_{PM}}§amp;gt;1$ and the second is for $E_{SI,P_{PM}}<1$ . By using a moving average with period of 3, we found that $E_{SI,P_{PM}}=1$ where *SI*=0.925. According to this, the first part of the data ( $E_{SI,P_{PM}}>1$ ) occurs when *SI* <0.925, in which a large change in SI responses with very low change in *P*_PM_. The second part of the data, occurs when *SI*>0.925, where the data presents low level of elasticity in terms of SI so $E_{SI,P_{PM}}⟶0$ , i.e. very small change in *SI* response in large change in *P*_PM_. This finding is used below. According to these results, and with regard to the PMTH results (Fig. 1), we can now conclude that the PMTH is a linear approximation of the phase transition pattern for closely related species.

### Part 3: the evolutionary scale – illustrations of the boundary between two evolutionary processes

**Fig. 3.**
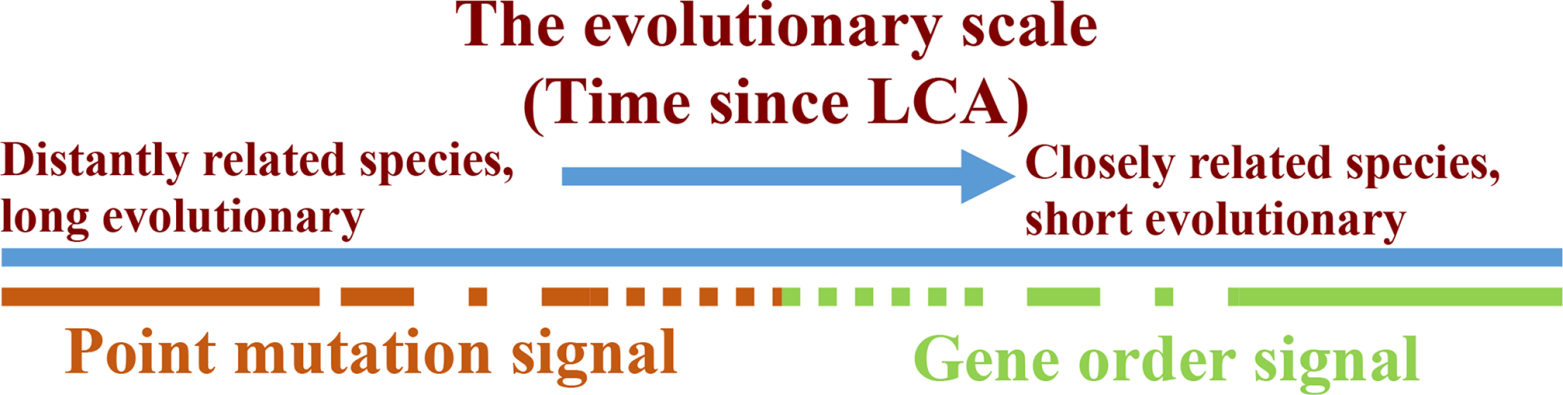
Graphic representation of the evolutionary distance line: here we demonstrate the evolutionary scale concept. The blue line represents evolutionary distance in terms of time since LCA (last common ancestor). On the right-hand side, we find strong gene order signal (green line), which characterizes closely related species. On the left-hand side of the evolutionary scale, we find the point mutation signal (red line), which supplies a stable signal among non-related species. We try to investigate the relationship between these two processes at the point of convergence between the green and the red lines – is there an overlap, a gap or a precise cross point? According to our findings, there is an approximate cross point, and in most cases, the gene order provides a reliable phylogenetic signal up to the genus level, while the point mutation process provides stable phylogenetic signal up from the genus level.

Now we present the evolutionary scale – the concept that the evolutionary time can be presented as a line, separated into two sections. In Fig. 3 the conventional geological time scale, time since speciation, moves from left to right. At the right-hand side, there are very closely related species, while distantly related species are present at the left-hand side. We can, theoretically, place each pair of species somewhere along this line, according to the height of their last common ancestor (LCA) in the evolutionary tree. As demonstrated in our previous work [28], closely related species, i.e. pairs placed in the right-hand side of the evolutionary scale, are phylogenetically analysed better by the SI approach, while at some point on the evolutionary scale, SI comes to saturation and data is better analysed by a point mutation-based approach. This concept is illustrated in Fig. 3. An important question, which rises from this concept is whether there is an overlap, a gap or a precise cross point between these two approaches. Fig. 2 demonstrates that a phase transition pattern arises from the data, which hints that there is an approximate cross point. If there was an overlap or a gap, we would find a linear stage in this graph. The phase transition point occurs where the point mutation signal overcomes the gene order signal, and we suggest it is the point where the first derivative of the phase transition function equals 1. Based on the least squares approximation approach,

(4) $$E\left(P_{PM}\right)=5*10^{-5}e^{8.3704SI}$$

(not to be confused with the elasticity term, which is noted as $E_{SI,P_{PM}}$ ), and we get that

(5) $$E{\left(P_{PM}\right)}^{\prime }=8.3704*5*10^{-5}e^{8.3704SI},$$

$E{\left(P_{PM}\right)}^{\prime }=1$ comes where *SI*=0.9293. In other words, the cross point arises where SI≅0.93, such that for SI values lower then 0.93, the point mutation process provides poor phylogenetic signal compared to SI, and gene order provides reliable phylogenetic signal. Beyond this value, SI arrives to saturation quickly, and the gene distance provides a more reliable phylogenetic signal. This result is very similar to the elastisity analysis, and will be used below.

### Part 4: from theory to practice: applications arising from the phase transition phenomenon – HGT detection and redefine the genus boundary

**Fig. 4.**
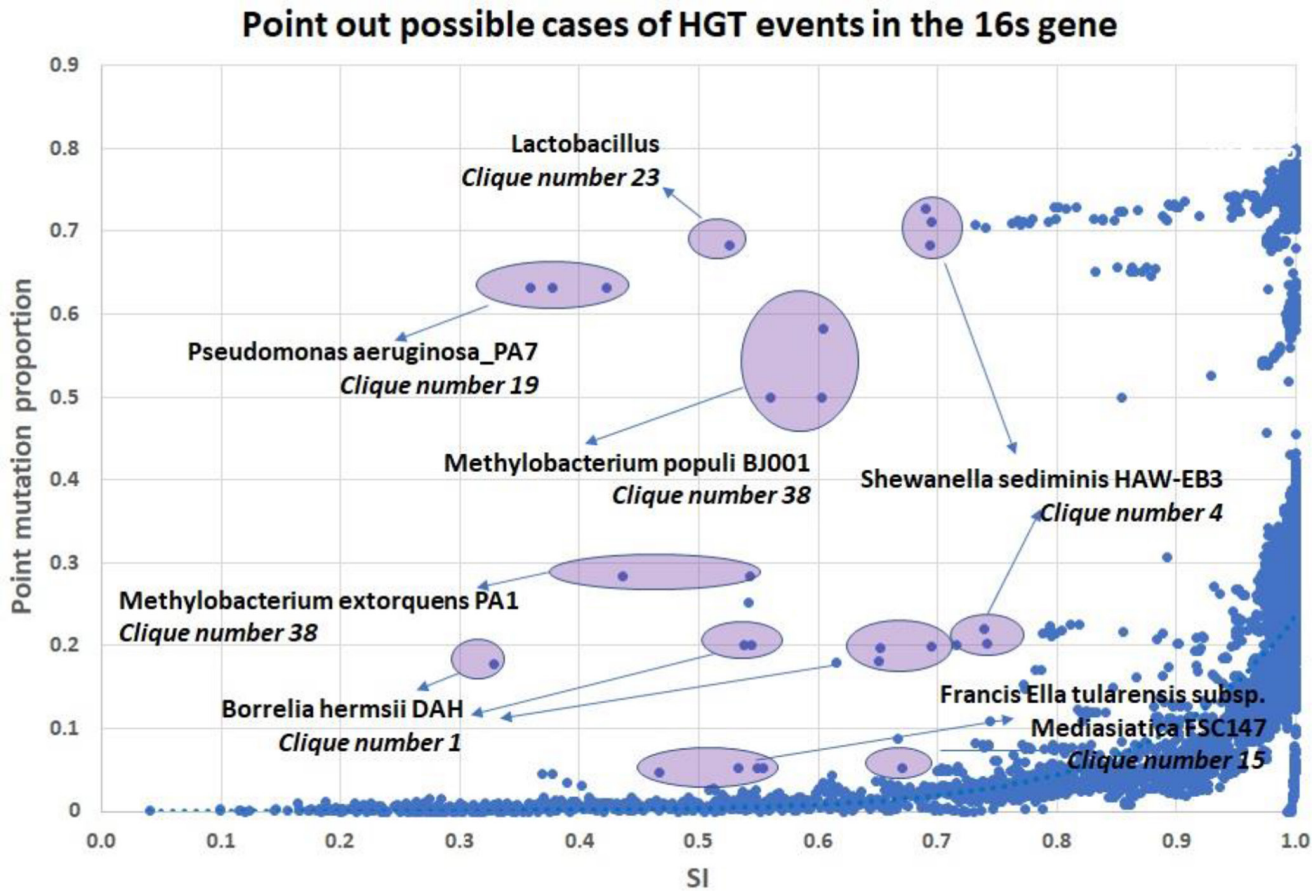
HGT detection based on the phase transition pattern. Here we plot the same graph as in Fig. 2, but we marked some of the outliers. Many of the outliers are clusters of pairs involved with one specific species, with other species of the same clique. We hypothesize that these outliers might hint rare cases of HGT events of the 16S gene.

Two important applications arise from the phase transition pattern we find between the gene order signal and the point mutation signal. The first is the ability to detect HGT events of the 16S gene. According to the ‘complexity hypothesis’, the 16S gene has not undergone HGT, so the 16S rRNA gene has been selected as a gold standard marker gene for prokaryotic classification [11]. But, as stated in the introduction, recent studies showed that this gene, and other housekeeping genes, might have undergone HGT events in some cases [12–15]. This phenomenon has an ecological, evolutionary and taxonomic importance, and here we suggest an automating approach for detecting such cases. In the graph shown in Fig. 2, there are a few outliers (~500 out of 50000 points), which do not match the phase transition pattern. In Fig. 4, we mark some of them, and we notice that most of these cases can be clustered to a few species. For example, in seven of these outliers the species *Borrelia hermsii DAH* from clique number 1 is involved. In these seven cases, this species is compared to a species belonging to clique number 1 and there is a higher than expected gene distance based on SI measurement. It is possible that such a cluster of outliers as a sign for a rare case of HGT event in the 16S gene in which *Borrelia hermsii DAH* acquired its 16S gene from a distantly related specie, outside clique number 1. Much deeper analysis is needed for such a statement, but this is the intuition for an HGT detection of the 16S gene we suggest here.

**Fig. 5.**
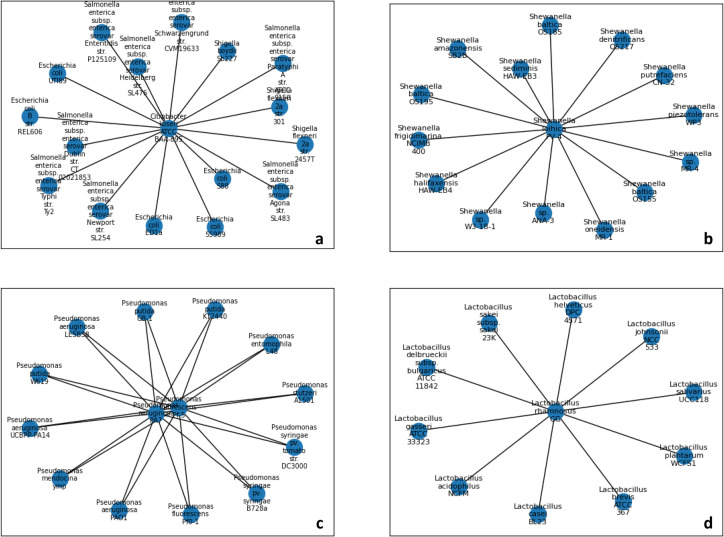
HGT detection based on analysis of outliers of the phase transition. Outliers in the phase transition graph (see Fig. 4) can hint an HGT event of the 16S gene if the same species is involved in many outlier points with its clique’s member. In order to find such cases, a graph was created for each clique, in which each species is a node and there is an edge between nodes if their *SI/P*_PM_ ratio is an outlier of the phase transition pattern (outlier is declared in the text if *P*_PM_ > 1.7 ∗ *E*(*P*_PM_)), i.e. more than two standard errors). If there is a prominent star in the graph we can assume this species receives its 16S gene from a distant species not belong to its clique. For example, *Citrobacter koseri* is a prominent star in its clique (clique 17, a), i.e. its 16S gene is much more distant than expected by its SI relation to its group member (*P*-value=1.5 * 10–16), and we conclude this species have a 16S gene, which was acquired from a distant species not belonging to clique 19. The same can be said for *Shewanella loihica* (b, clique 17, *P*-value=1.5 * 10–12), the pair *Pseudomonas aeruginosa* and *Pseudomonas fluorescens* (c, clique 19, *P*-value=0.0002), and *Lactobacillus rhamnosus* (d, clique 23, *P*-value=0.00095).

**Fig. 6.**
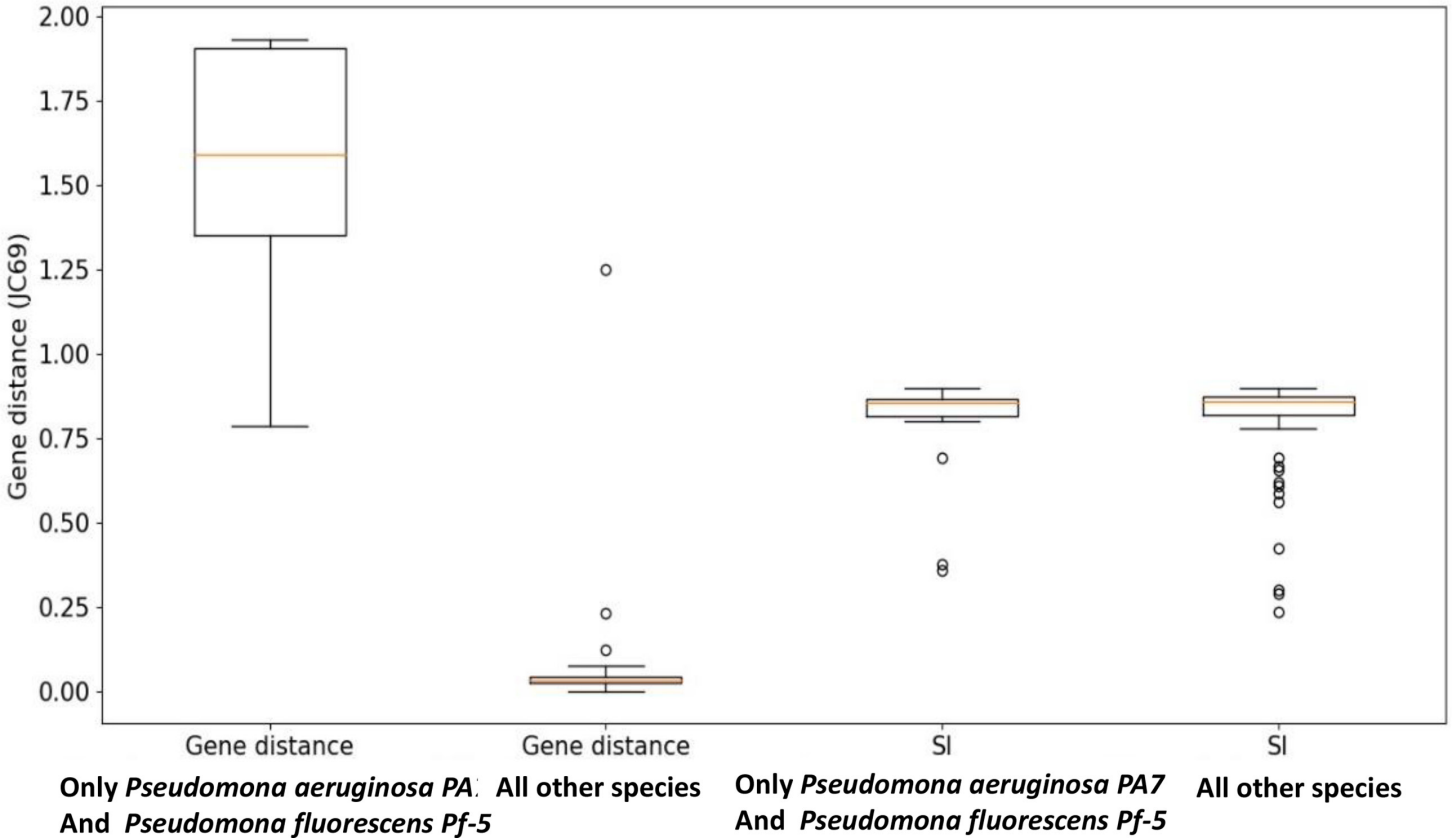
Boxplot of gene distance and gene order distribution of all species with a focus on two outliers. From left to right: the first column presents the gene distance distribution of all connections between the species *Pseudomonas aeruginosa* to all other species in its clique (clique number 19), and also the species *Pseudomonas fluorescens* to all other species in clique 19. The second column presents the same measure for all connections in clique 19. The third column presents SI distribution of all connections between the species *Pseudomonas aeruginosa* and *Pseudomonas fluorescens* to all other species in clique 19, and the last column presents SI distribution for all connections in clique 19. It can be seen that the two *Pseudomonas* species present, unexpectedly, long gene distance to their relatives in the clique, which is not correlates with their SI measurement. This may hint these two species acquired their 16S gene out of the clique.

For a more systematic analysis, we use the following approach. For each clique from [28] we created a ‘clique-outliers graph’, in which the clique’s species are the nodes and there is an edge between two nodes if the gene distance between them is an outlier according their gene order. We defined outliers if $P_{PM}>1.7*E\left(P_{PM}\right)$ , since 2 standard errors of $P_{PM}$ are found to be $1.7*E\left(P_{PM}\right)$ . Next, we looked for stars in these clique’s graph, i.e. graphs in which one species is connected to many other species, more than can be expected by chance. We identify stars based on the probability to get such a star or a more extreme star (e.g. one node with similar or more edges) in a random graph with the same number of edges and nodes, with a threshold of 5 %. Fig. 5 presents four such star-graphs, which hint for an HGT of the 16S gene among five species: Lactobacillus rhamnosus GG, *Shewanella loihica PV-4*, Citrobacter koseri ATCC BAA-895, *Pseudomonas aeruginosa PA7* and *Pseudomonas fluorescens Pf-5*. The two-last species (* Pseudomonas *) present interesting pattern. Both present typically SI values in respect to others members of clique 19, but high values of gene distance to other species of the clique. Among the 91 possible pairs of cliques number 19, 25 of them are with gene distance >0.5, and in all these cases these two species are involved. While the average gene distance value of clique number 19 is 0.46, the average gene distance value of pairs contains one of these two species is 1.6, and the average gene distance of pairs do not contain these two species is 0.053. The value of gene distance between these two species is 0.78. These might be explained if these two species acquire there 16S gene outside the clique. Fig. 6 is another representation of the uncorrelated measurement of gene distance and gene order of two species, which may hint 16S acquisition fom a distant source.

In a very interesting way, based on the phase transition pattern, we can suggest an analytic and objective method for genus boundary definition, using the point where the curve of the phase transition's first derivative is equal to 1 ( $E{\left(P_{PM}\right)}^{\prime }=1$ ). At this point, there is equal quality of phylogenetic signal provided by gene distance and gene order, and these two evolutionary clocks tick at the same rate. This point occurs where SI=0.93 . This can be reinforced based on the result that the elasticity comes to 1 at the same point. This is an objective way for solving the genus classification problem, based on the phase transition pattern between the gene order and the gene distance signals.

The second application that arises from the phase transition pattern, is a suggestion for redefinition of the genus boundary. Species within a genus are supposed to be somehow similar, but, as stated in the introduction, there are no objective criteria for grouping species into genera [19], since genus definition is not based on an analytical measurement and it is subjected to bias according to researchers’ background. We noted before about the known relation between mutation and recombination [34] inside the genera level, and based on the phase transition pattern, we can suggest an analytic and objective method for genus boundary definition, using the point where the curve of the phase transition's first derivative equal to 1 ( $E{\left(P_{PM}\right)}^{\prime }=1$ ). At this point, there is an equal quality of phylogenetic signal provided by gene distance and gene order, and these two evolutionary clocks tick at the same rate. This point occurs where SI=0.93 . This can be reinforced based on the result that the elasticity comes to 1 around this point. While in our previous study we set the threshold for cliques to be SI=0.95 , based on some trial and error, here we found a very similar threshold value, based on the analytic objective criterion of the phase transition point. As previously been shown [28], the cliques produced by this threshold are similar to the classical taxonomy and the genus level. A theoretical clique-like concept for genus definition suggested by [19], but the author notes about the absence of an appropriate measure for fully objective ranking criterion for species. Here, we offer an objective way for solving the genus classification problem, based on the phase transition pattern between the gene order and the gene distance signals.

## Discussion

This is a follow-up work of [28] and [25], from an evolutionary point of view. Here, we analyse and compare between two evolutionary clocks. The first is based on the point mutation process, which is measured by the probability of point mutation, *P*_PM_, using the Jukes Cantor 69 model (JC69) [32], and this serves as a measurement for gene distance. The second is based on genome rearrangement processes, such as HGT, which has a major impact on gene order, measured by calculating the probability for HGT event per gene (or any other genome rearrangement event), based on SI. The relation between these two evolutionary clocks, or processes, was experimentally investigated many times, for example in [34], and it was suggested that these two processes are mechanistically associated or that one process provokes the other [37]. The importance of this ratio is in estimating of the relative roles of HGT and recombination in one hand, and point mutation in the other hand, in the generation of new alleles, as it clarifies how organisms evolve [38]. This study is based on 1133 species, mainly bacteria, from the eggNOG database for gene order measurement and from RDP database for the 16S gene distance measurement. Here we found that the two evolutionary clocks present two different basic patterns. One pattern is among closely related species (i.e. inside genera level), in which an approximate linear relation between these two clocks occurs, i.e. as gene distance increases, gene order increases, in accordance with some constant. More specifically, we found that gene order signal is about seven times stronger than the point mutation signal ( $\frac{1}{0.1394}≅7$ , where 0.1394 is the regression coefficients), i.e. increasing of gene distance by one unit corresponds to increasing of 7 units of gene order. In other words, among closely related species, gene order signal is dominating gene distance signal. The second pattern was found for all species, closely and distantly related species, and a phase transition pattern between these two measurements was found. We analyse this pattern and detect that the phase transition point occurs where SI≅0.93 . In our previous work [33], we developed a statistical model, which can be used to translate SI to the number of recombination events [i.e. to a real distance function, equations (1,2)] [33]. Based on this model we can see that the point of phase transition occurs where p≅n (since this leads to $E\left(SI\right)≅0.93$ ), which means that at this point each gene in the genome undergoes, on average, one translocation. For example, if $E\left(SI\right)=0.93,k=10$ (this value of k leads to ${E\left(SI\right)}^{\infty }=0.98$ ), we get $\frac{p}{n}=1.008$ , where p is number of events and *n* is genome size. Next, we declare the term ‘evolutionary scale’, which represents the time since divergence, and we separate this line into two parts: the left part, for long time since LCA pairs of species, is where the point mutation process provides more accurate phylogenetic signal, and the right part, for recently LCA pairs of species, where gene order provides the most meaningful signal. The phase transition pattern indicates that an approximate meeting point exists between these two parts, such that the phase transition point occurs where the point mutation signal overcomes the gene order signal and vice versa. Two main important meanings arise from the phase transition pattern. The first, is the ability to detect an HGT event of the 16S gene, based on the outliers' point of the curve. This is an important and novel task, since housekeeping genes were for a long time perceived as resistant to HGT events, but today we know that there are rare such cases, as explained in the introduction. We present the star-finding approach for detecting such cases, and also present an example of the great differences between the distribution of gene order and gene distance of two such cases.

The second application that arises from the phase transition pattern, is a suggestion for redefinition of the genus boundary. Species within a genus are supposed to be somehow similar, but, as stated in the introduction, there are no objective criteria for grouping species into genera [19], since genus definition is not based on an analytical measurement and it is subjected to bias according to researchers’ background. We noted before about the known relation between mutation and recombination [34] inside the genera level. Here we use this relation, and show how the point of the phase transition, in which each gene undergoes on average one translocation event and the two evolutionary clocks tick at the same rate, can serve as an objective ranking criterion for the genera level. While in our previous study we set the threshold for cliques to be *SI=0.95*, based on some trial and error, here we found a very similar threshold value, based on the analytic objective criterion of the phase transition point. As has previously been shown [28], the cliques produced by this threshold are similar to the classical taxonomy at the genus level. A theoretical clique-like concept for genus definition suggested by [19], but the author notes about the absence of an appropriate measure for fully objective ranking criterion for species. Here, we offer an objective way for solving the genus classification problem, based on the phase transition pattern between the gene order and the gene distance signals. This systematic approach has the potential to overcome the disadvantages of the current approaches, which based on some subjective criteria and the lack of a theory-based concept of what properties a genus should have.
